# Trends and Drivers of Change of Pastoral Beef Production Systems in a Mediterranean-Temperate Climate Zone of Chile

**DOI:** 10.3390/ani9121135

**Published:** 2019-12-12

**Authors:** Paula Toro-Mujica, Raúl Vera, Einar Vargas-Bello-Pérez, Pablo Pinedo, Fernando Bas

**Affiliations:** 1 Instituto de Ciencias Agronómicas y Veterinarias, Universidad de O’Higgins, San Fernando 3070000, Chile; 2 Departamento de Ciencias Animales, Facultad de Agronomía e Ingeniería Forestal, Pontificia Universidad Católica de Chile, Av. Vicuña Mackenna 4860, Casilla-306 Santiago, Chile; rverai@uc.cl (R.V.); fbas@uc.cl (F.B.); 3 Department of Veterinary and Animal Sciences, Faculty of Health and Medical Sciences, University of Copenhagen, Grønnegårdsvej3, DK-1870 Frederiksberg C, Denmark; evargasb@sund.ku.dk; 4 Department of Animal Sciences, College of Agricultural Sciences, Colorado State University, Fort Collins, CO 80523, USA; pablo.pinedo@colostate.edu

**Keywords:** beef cattle, comparative evolution, farms continuity, resource use, typology

## Abstract

**Simple Summary:**

Livestock production systems show modifications over time due to both internal and external variables. The relationships between internal variables allow the definition of typological groups of production systems. Knowing the typological groups and their temporal evolution allows the identification of continuity strategies at the private and public levels. With this aim and using data from livestock surveys conducted in 2009, 2011, 2013 and 2015, four typological groups of beef production in a Mediterranean-temperate climate zone of Chile were identified. The typological groups differed in size, productive orientation, breeds used, and response to changes in external variables, among other aspects. These groups require different continuity strategies, which include, their sustainable intensification and the need to focus on pastoral production using low external inputs to enhance the production of “natural” beef for high-value niche markets.

**Abstract:**

The present study used surveys of the cattle sector over the period of 2009–2015 to develop a typology of cattle farms to evaluate their evolution over time and to identify variables that may be associated with systems’ adaptive changes and continuance. Four groups of farms were defined using multivariate analyses as follows: Group I are small calf-cow operations using non-specialized beef breeds; Group II is similar to Group I but employs specialized beef breeds; Group III is dedicated to finishing cattle, and Group IV are larger farms (>1000 animals) with a complete cycle of breeding and fattening. In general, beef cattle production in the temperate—Mediterranean Southern Zone of Chile is declining in response to the opening up of the economy that allows for ample imports, the high opportunity cost of land, and recurrent droughts associated with climate change. Current policies and regulations have modified farms’ businesses models depending on their ease of access to markets, farm size and financial capacity. The defined groups require different development paths and strategies. Sustainable intensification is an alternative strategy for farms in Group I and II, particularly if they were to contract the finishing stage of their cattle with Group III farms. In contrast, it is suggested that Group IV farms concentrate on pastoral production using low external inputs to enhance the production of “natural” beef for high-value niche markets, with positive externalities.

## 1. Introduction

Chile has a wide diversity of agricultural production systems associated with its variety of soils, climate and geography. Livestock are present in all agricultural production systems in varying degrees, a trait that is shared throughout Latin America and the Caribbean (LAC) [1].

Chile accounts for only 0.3% of global beef production [2]. The high quality of the meat produced in pastoral systems in southern Chile enables its positioning in niche markets [3]. On the other hand, the contribution of beef production systems to food production and environmental conservation is threatened by climate change and by the international demand for energy, food and biofuels [4,5,6].

In 2007, Chile had 301,269 farms occupying 50% of the country’s agricultural land [7]. Ninety-two percent of these properties qualified as ‘family farms’. Cattle production occurred on 45% of farms, concentrated mainly in Chile’s Southern Zone which, in 2007, accounted for 75% of the national herd or approximately 2.6 million head of cattle [8]. Contrarily to the majority of LAC, Chile’s livestock sector only accounts for 16% of the agricultural gross domestic product [9].

Farm surveys revealed a decrease of 19% in the number of cattle between 2007 and 2015 [10]. Furthermore, Chile’s Southern Zone has been affected by increasing drought in frequency and severity [11] and for the existence of more profitable alternative land uses, a common scenario worldwide [12,13]. Relatively inexpensive beef imports have also increased by 14% annually [14]. In response to these trends, the government has promoted increased beef production efficiency and the search for value-added beef products, supported by the implementation of cattle traceability, investments in soil and pasture reclamation, more efficient irrigation structures, and improved marketing channels [15]. 

Generally, beef systems of Southern Chile are extensive, relying on native and sown pastures and cereal stubbles. Strong seasonality of forage production (60% in spring) [16] forces producers to supplement animals with alfalfa or red clover hay, and wheat straw. However, there is large variation between farms in management, land use, and producers’ attitudes towards the adoption of technology [17,18,19]. 

The diversity, existence, and evolution of livestock systems is associated with the prevailing socioeconomic context and agricultural policies and with regional and local factors that determine production potential and access to inputs and markets [20]. Within the agricultural sector of Chile, the opening up of the economy, free trade agreements, and processes of modernization have forced noncompetitive farms to abandon the cattle sector [21] or, in order to remain viable, increase production efficiency [22], a scenario common to many developing and developed countries alike. 

Farm heterogeneity in area, number of animals, breeds, productive system, and forage crops presuppose the existence of a variety of strategies for the adaptation of farming systems to changing climate and economic conditions [23]. Typology of livestock systems to identify relatively homogeneous farm groups would facilitate the development of targeted agricultural policies to help to ensure the viability of the Chilean livestock sector. Numerous multivariate statistical methodologies have been used to develop farm typologies (e.g., [24,25,26,27,28]). 

In this context, our objective was to use information collected in comprehensive livestock surveys to characterize beef production systems in the Mediterranean and temperate Southern zone of Chile over the 2009–2015 period, assess their evolution over time, and hypothesize the reasons for the observed changes as well as the strategies of adaptation and continuity in relation to farm types.

## 2. Materials and Methods

### 2.1. Study Area

**Figure 1:**
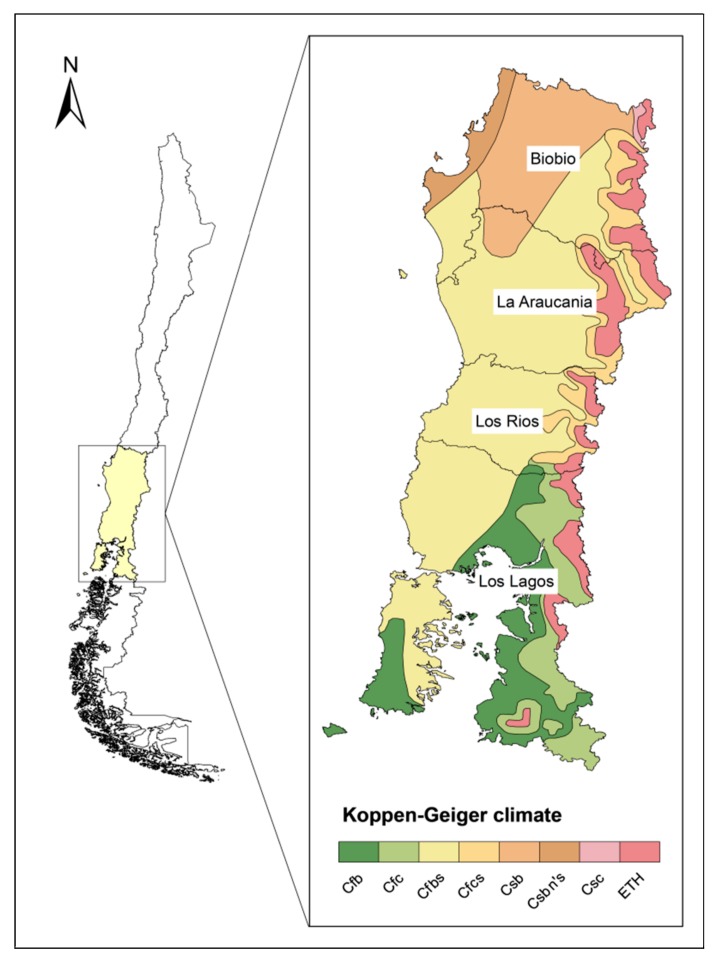
Koppen–Geiger climates in study area.

The main beef producing area of Chile is the Southern Zone, comprised of the Biobio, Araucanía, Los Rios and Los Lagos regions, located between latitudes 36°00′–44°03′, longitude 70°51′06″, and the Pacific Ocean, for a total of 135.924 km^2^. The northern extreme of the zone has a warm temperate climate (Csb), with rainfall ranging between 799 and 900 mm, mostly in winter. The climate of the remainder is humid temperate (Cfsb), receiving a wide range of precipitation that reaches 1500 mm in Los Lagos (Figure 1). The mean annual temperature is 12–13 °C in the northern, and 8–10 °C in the southern limits [29]. These conditions allow for year-round grazing on native and improved rangelands and sown pastures composed of a variety of Mediterranean and temperate species described by [30]. Depending on the climate zone (Csb or Cfsb), yearly dry matter yields range from 4 to 13 ton per ha [31].

According to the last Agricultural Census [8], the study area houses 69.8% of the bovine farms and 58.2% of the stock of cattle in Chile.

### 2.2. Data Sources

A series of biennial surveys of the livestock sector has been conducted since 2009 by INE [32,33,34,35] and ODEPA (Agricultural Policy and Studies Office) to characterize Chile’s beef production systems. The farms sampled represented more than 20% of farms stocked with ≥10 head of cattle in the 2007 Agricultural and Forestry Census. 

Although the farms with less than ten cattle heads correspond to a high percentage of the total farms, the livestock surveys do not consider them, so they were not incorporated in the study. The main agricultural activities in farms with less than 10 animals include cereal crops, fowl, and milking of dual purpose cattle, whereas beef cattle constitutes only a complementary, occasional and minor component that contributes little to farm income.

Sample size was determined for each of the four regions (Biobio, Araucanía, Los Rios and Los Lagos regions), in proportion to the number of animals in the region and the Southern Zone, with a relative error of 5% [36].

The number of farms surveyed was 2592, 2534, 2877 and 3140 for 2009, 2011, 2013 and 2015 respectively. More recent information is not available, although a new census is tentatively scheduled for 2020. The surveys included 148–191 closed questions divided into eight sections as follows: I. Farm identification; II. Farm description and activities; III. Farmer characteristics; IV. Employment; V. Land use and pasture management; VI. Cattle; VII. Dairy production and; VIII. Beef production. Table A1 provides an example of the survey format and some typical closed questions. Minor differences in survey format were apparent between years; however, 72 variables were common to all, 14 of which were qualitative (some examples in Table A1). These and 43 additional variables (calculated from the original variables), were used in the analyses [37,38] (Table 1 and Table 2). The surveys’ samples were randomly stratified and proportional to herd size, according to census data. Small, medium and large farms carried 50–99, 100–299, and >300 head respectively. Trained agricultural professionals carried out written surveys over a 4-month period (July–October) of each year. Information was reviewed and entered into a regional information system and subjected to various automatic validations. When discrepancies arose, the interview was repeated. Data were then compiled at the national level and compared with data from national agricultural census and other sources.

### 2.3. Statistical Analyses

Several data management and technologies were used to describe and analyze the temporal evolution of the existing farming systems, including the original and calculated variables related to farm size, land use, and stocking rate. Descriptive statistics were calculated and depending upon the homogeneity of the variances (Levene test), multiple comparison of means (Student–Newman–Keuls) or Kruskal–Wallis [23] were carried out as described by [39]. Qualitative variables were analyzed with contingency tables and chi-square tests (X^2^) [40,41]. A multivariate analysis of all available variables was carried out using the data from the 2009 survey considered as the base scenario.

All the farms present in the 2009 survey were used for analyses following the methods of [42], also used by [39,43,44]. Initially, quantitative variables were characterized with descriptive statistics, their coefficient of variation (CV), and the correlations between them. Variables with a CV less than 60% were discarded. In the case of highly correlated variables (≥90%), those that had the lowest number (one or two) of high correlations with the remaining variables were similarly discarded. Variables without correlations greater than 70% with some other variable were also eliminated, followed by an assessment of multiple correspondences, for which quantitative and qualitative variables were used as in [45]. The Chi-square test was used to determine the relationship between quantitative and qualitative variables and to select between a multiple correspondence and a factor analysis [24]. Principal components with varimax rotation were extracted for the factor analysis [38,40]. Sampling adequacy was assessed with the Kaiser–Meyer–Olkin test and Bartlett’s sphericity test [46]. Factors with an eigenvalue larger than one were retained [47,48]. The magnitude of factors for each farm was used for a hierarchical analysis of conglomerates [26,49]. Five grouping methods were tested, namely, the closest neighbor, farthest neighbor, grouping of centroids, grouping of means, and the Ward method, using the Euclidean distance, square Euclidean distances and the Manhattan distance [50]. Selection of the most appropriate method was based on the analysis of dendograms and clusters distances [51]. The number of clusters in each dendogram was chosen based on the distances, and the number of clusters accepted was that in which the graph of the clustering distances showed an inflexion [52]. Lastly, a discriminant analysis was carried out to evaluate the appropriateness of the groups [39,53]. Once the groups and the methodology for obtaining them were defined, factor coefficients were extracted. With these coefficients, the factorial scores of each factor and each farm in the following years were calculated. Subsequently, farms were assigned to groups using a discriminant analysis. The within-group evolution of farming systems, as well as the importance of each group in the total farms was analyzed, followed by the calculation of descriptive statistics for each group, and year within group.

Once the groups were defined, their evolution was based on the comparison within the group of the selected variables through analyses of variance and multiple comparisons of means (Student–Newman–Keuls) or Kruskal–Wallis test and boxes and whiskers graphs, depending on the homogeneity of variance and normality test. The level of significance used was 0.05. All statistical analyses were carried out with SPSS 11.5 [54].

Contingency tables were calculated for the qualitative variables using the X^2^ test and the adjusted residuals were used to assess differences in the response variables. The definition of the adaptation strategies and their relationship with the drivers of change were identified through the identification of the particularities of each group, that is, based on the comparison between groups through the aforementioned statistical analyses.

## 3. Results

### 3.1. Land Use and Farm Descriptions

The preliminary analyses showed large between-farm variability for several variables (Table 1), a feature that is common to beef production systems elsewhere [5,6,55]. Average farm size decreased significantly over the study period, whereas the stocking rate experienced a decrease in 2009–2011 and was stable thereafter (Table 1). Sown and improved pastures also decreased, whereas naturalized pastures increased (Table 1). Similarly, the area sown to annual forages (oats, annual ryegrass, maize) decreased between 2009 and 2011 (Table 1).

### 3.2. Definition of Production Systems

Each of the 58 quantitative variables available had a CV larger than 60%, but 38 were eliminated due to the absence of information on 25% of the farms, or due to the size of correlations between them. To analyze the relevance of a multiple correspondence analysis, the remaining 20 variables were grouped in 3 or 5 categories, depending on their variability. Chi-square tests revealed low relations among variables, and therefore, a factor analysis was used instead of a multiple correspondence analysis. The Kaiser–Meyer–Olkin test reached a value of 0.7, deemed adequate, and the Barlett sphericity test was significant (*p* < 0.01) [56]. Five principal components accounted for 66% of the variability. The percentage variability accounted for each one, and the relationship of each component with each variable is shown in Table 3.

### 3.3. Typology of Pastoral Beef Production Systems

Beef production systems were successfully separated by the analysis of principal components (PC) that led to defining 5 PC. The first one, PC1, showed the importance of herd composition with breeding herds having a lower relative weight, indicating the overriding importance of growing and fattening cattle in these systems. As expected, dimensional variables such as farm size and number of animals were also extremely important (PC2) in grouping production systems. A concurrent finding was the importance of supplementary annual forages, such as oats, annual ryegrass, maize and others (PC3) indicating their prevalence in the larger properties. Therefore, the coincidence of larger farms, newer cattle breeds (coinciding with the weight received by cattle breeds in PC5), and the use of supplementary forage crops point to the search for efficiency and higher incomes in the previously mentioned context of high input prices and increasing competition with imported meats. On the other hand, smaller farms resorted to relying relatively more on pastures and less on crops but were able to do so using lower stocking rates (PC4).

**Figure 2:**
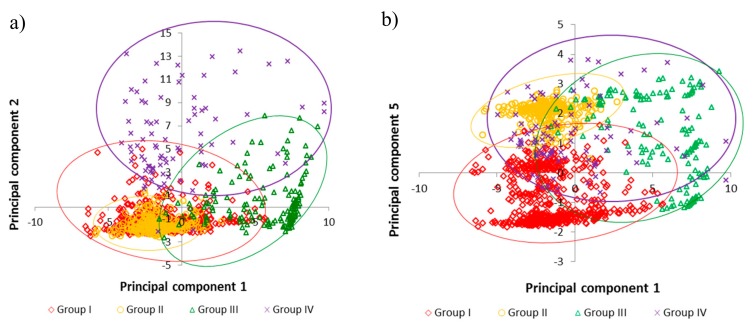
Classification of farms: panel (**a**) according to PC1 and PC2; panel (**b**) PC1 and PC5.

**Figure A1:**
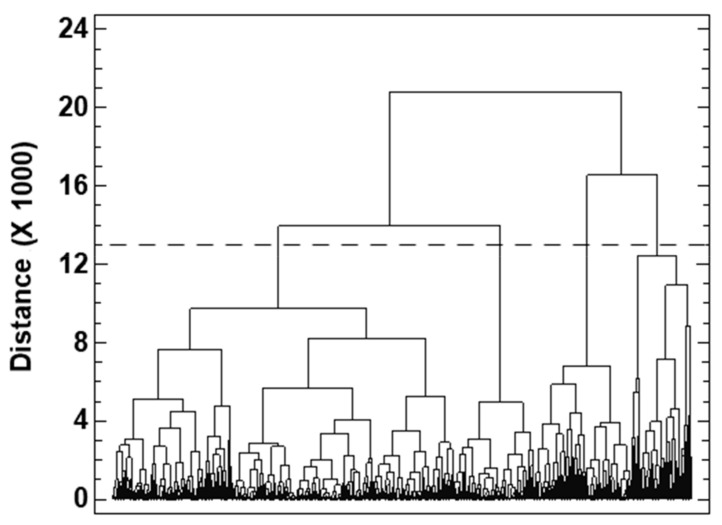
Dendrogram using the square euclidean distance grouping method.

The clustering method that gave rise to the better defined dendrograms was the Ward method with the squared Euclidean distance (Figure A1). These analyses, together with ANOVA analyses and multiple comparison of means, verified the adequacy of establishing four farm groups. Discriminant analyses correctly assigned 91.6% of the farms to the original groups. As an example, Figure 2 shows farms distribution in 2015 within groups for PC1 vs. PC2, and for PC1 vs. PC5, respectively. Figure 2b demonstrates that PC5 is essential to differentiate groups I and II given the similarities in a large proportion of their characterization variables (Table 2 and Table 4). The description of groups, based on their differences and similarities shown in Table 2 and Table 4, provided the background to assess the evolution of beef systems over the 2009–2015 period, and the extent to which several variables influenced the systems’ resilience and robustness that underlie various strategies of adaption over time. 

The description of the groups is as follows:

Group I. Cow-calf systems using non-specialized breeds.

This is the largest group, accounting for 59% of the farms surveyed. Farm size ranges between 300 and 700 ha, and together with Group II, they have the smallest herds. They rely on native and improved pastures, whereas annual forages are scarce. On average, 60% of the farm area is allocated to cattle where dual-purpose animals predominate. These farms do not fatten animals and management is minimal, as reflected by the scarce subdivisions and the little use of electric fences, the measurement of available forage, and the lack of regular soil analyses (Table 4). Labor productivity is also low. These characteristics coincide with poor financial management, the lack of investments and low input by agricultural advisers. Animals are sold in informal markets (direct sale between farms) or in agricultural fairs, a fact that reinforces the cow-calf orientation of these small farm systems.

Group II. Cow-calf systems using specialized breeds

Farm size is slightly larger than in Group I, but crops constitute an important land use in this group. Cow calf systems are important, but contrarily to Group I, beef breeds and crosses predominate. Herd size in this group is similar to that of Group I, but stocking rates are lower due to the predominant use of native pastures, with a small contribution of other forage resources, and much of the farm land is dedicated to non-livestock activities (e.g., forestry and orchards). In consequence, management is extensive and labor use is small. Animals are sold either in fairs or through intermediaries. Contrarily to Group I, practices such as soil analyses likely related to cropping activities, and the use of electric fences is common, and the level of capital investments resembles that of Group III.

Group III. Cattle finishing using specialized breeds

Although farm size is similar to the previous two groups, herd size and stocking rates (1 LU/ha) are larger due to the inclusion of 15.9% of sown pastures, 37.1% improved pastures and regular use of annual forages such as ryegrass, oats and maize silage. On the other hand, annual crops cover only 25.5% of the farm. The main activity of these farms is fattening pure breed or crossbreed steers. The use of supplementation increases the amount of labor. Soil analyses and the use of electric fences for subdivisions are common. The large volume of production is associated with the sale of animals in private fairs, and some are sold to slaughter plants.

Group IV. Farms with a complete cycle

These much larger farms and ranches are found mostly in Los Rios and Los Lagos regions. Given their size, a complete cycle of production from calf production to fattening of steers is generally found. Animals graze on a variety of native, improved, and sown pastures, but supplementation with maize silage is important. The latter predominates in the finishing phase of steers, either through conserved forage or soiling. The main breed is Aberdeen Angus, but other breeds and crosses are also found. This group makes more intensive and efficient use of labor, which results in a lower labor requirement per head (0.9 permanent workers per 100 animals). Management practices are frequent, such as the performance of soil analyses, the use of electric fences, and frequent measurement of available forage. Similarly, the use of agricultural and management consultants is common, as are larger investments on pastures.

**Figure 3:**
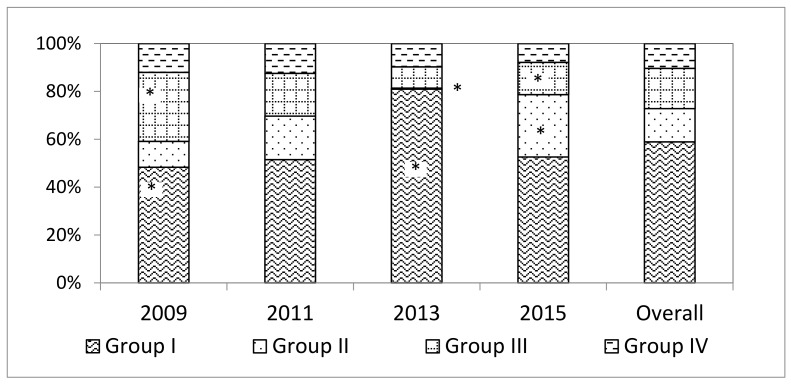
Percentage distribution of groups according to year of survey. * Percentages significantly different from the expected value (*p* < 0.05).

**Figure 4:**
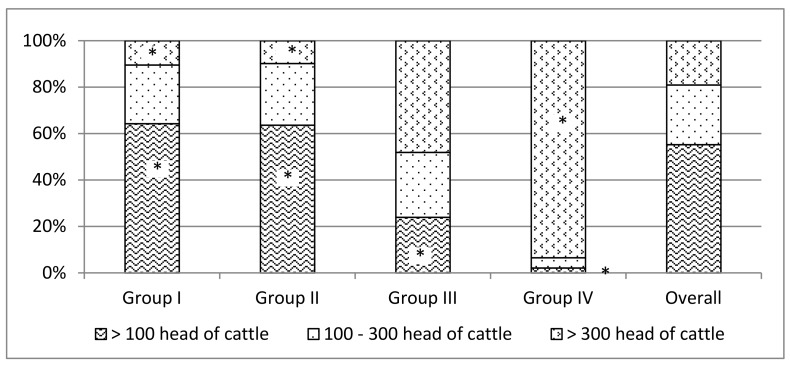
Dimensional distribution of farms by group. * Percentages significantly different from the expected value (*p* < 0.05).

Figure 3 shows the temporal dynamics of the beef systems studied. Group IV was the most stable group (Figure 3 and Figure 4), probably owing to farm size and to their peculiar mix of land use types that includes a significant contribution of crops (Table 2).

## 4. Discussion

The decrease in the size of the beef herds, together with a diminished number of beef production farms accounted for the 19.3% decrease in the regional herd but followed the national trend regarding the diminished national stock of cattle. Unexpectedly, improved pastures also decreased whereas naturalized pastures rose, as shown in Table 1. Low farm profitability, compounded by increases in prices of purchased inputs, may have led to the substitution of grazing lands for supplementary crops on the more fertile soils [57,58,59], a phenomenon also observed in other countries [60,61,62]. A parallel and significant increase of non-traditional cattle breeds such a Wagyu, Fleckvieth, and Belgian Blue, and crossbreds between these breeds took place [34,35], probably seeking higher revenues in a context of increasing input prices and competition with imported beef. On the other hand, the past common practice of fattening dairy calves decreased given the full ban on the use of growth promoting anabolic products for cattle [63]. This finding is highly significant given the historical reliance on dairy calves for beef production [64], that contrasts markedly with the situation observed in neighboring countries and points to an increasing specialization of at least part of the sector.

Marketing channels changed with a decrease in the number of cattle fairs and an increase in processing in slaughter plants, possibly associated with increasing beef exports to high value markets in Asia, the EU and the US [65]. Overall, investments in beef systems remained relatively stable, with the exception of a rise in the use of soil and pasture reclamation credits facilitated by the Agriculture Ministry [66] which, nevertheless, did not compensate for the generalized decrease in improved pastures. Investments in marketing and management consultants were very scarce, whereas the use of agricultural and veterinarian consultants was recorded in 30% and 45% of the farms, respectively. Given the expected impact of management and of agricultural and veterinarian advisers on farm income [16,67,68], it would be desirable that their use be increased.

As indicated above, there was a marked decrease in Chile’s cattle stock that fell from 3.85 million head in 2009 to 2.74 million in 2015 [2]. Given all of the above changes, it is hypothesized that farms that abandoned beef production were those that had the best soils and the least amount of native grasslands, whereas farms that kept beef cattle modified their feeding strategy and minimized costs by reducing pasture improvement.

The changes experienced by the farm groups over times revealed different dynamics. Thus, the relative stability of Group IV contrasts with the more pronounced changes experienced by the rest of the farms and it is hypothesized that these larger farms and more diverse production system were better buffered against changes in the economic and market environments. Nevertheless, there were also some commonalities across farm groups. In effect, examination of the within-group evolution between surveys revealed a number of general trends, as well as trends that are group-specific. In general, there was a decrease in the number of permanent workers, an increase in the percentage of native pastures, and a decrease of sown forage crops. Group I maintained stable stocking rates in spite of lower percentages of sown and improved pastures, and forage crops. Farm and herd size decreased, and the breed composition of the herds changed to a variable degree (Table S1), with a noticeable increase in Overo Colorado. There were no major changes in marketing channels, although the contribution of fairs diminished and that of intermediaries increased. Group II experienced a significant decrease in the number of farms during 2013 (Figure 3), probably due to the falling prices of beef during 2012 and 2013 and to a long drought in 2013. Some of these farms moved to Group I (Figure 3), whereas others ceased producing beef animals. Farms remaining in Group II corresponded to those of larger size, which led to changes in the average values of variables characterizing this group, such as average farm size. Native pastures increased, both in absolute and relative terms if data for 2013 is ignored, with a concomitant decrease in other land uses. The number of breeding cows increased, whereas that of other categories fell together with cultivated crops, which suggests a degree of specialization in breeding activities. In common with other groups, the number of permanent workers decreased, and their efficiency increased. There was a significant jump in the number of veterinary and management consultations (Table S2).

Group III showed little variation in stocking rates between years, despite an increase in native pastures in terms both of absolute and relative values, and a decrease in improved and artificial pastures. Farm size tended to increase but it was not accompanied by the number of animals, whereas the contribution of Aberdeen Angus to the herds increased. Previous practices such as the use of electric fences and soil analyses decreased, but there was a large increase (15 percentage units) in the measurement of forage availability, possibly associated with a rise in the use of consultants. Several of these changes may well be related to the intensification of grassland usage.

As previously indicated, farms in Group IV were the most stable in terms of size and animal numbers, but with a relative increase in the percentage of cows and decreased number of steers. Undoubtedly, farm size and available resources contributed to the larger resilience of these systems that were able to buffer changes in relative prices and possibly, in climatic parameters. As was the case for Group III, farms in this group diminished the use of soil analyses and electric fences and beginning in 2013, they increasingly relied on beef factories as the main marketing channel.

Considering the increasing demand for water, Chile’s government implemented a number of laws (e.g., Law N. 18450) that subsidize studies, construction and reclamation of irrigation related infrastructure. A quarter of the 11,017 studies financed over the period 2007–2015 were located in the study region. The number of farms in the region amounts to 161,594, which implies coverage of 1.7% of the properties [8,69]. In contrast, 27.2% farmers indicated in 2015 that the main limiting factor for beef production was water availability (Table 5). It is apparent that farms will continue to rely heavily on incentives to develop irrigation infrastructure and water conservation if they want to adapt their farms to the observed trends in climate change. A second policy tool is related to legislation related to reclamation of degraded soils (SRDS; Laws N. 19604 and 20412; [66,70]), that encourages improved soil management practices, fertilization, and the establishment of vegetative cover crops. A total of 519,191 ha benefitted from some of these practices during 2003 and 2007, but available surveys do not provide further evidence. It is possible nevertheless, that the marked increases in pasture-related investments registered in 2011 were associated with those incentives (Table 1).

**Figure A2:**
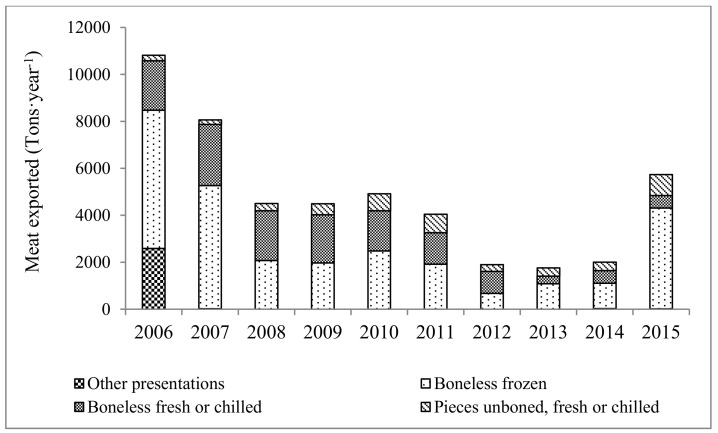
Chilean beef exports (ton·year^−1^). Source: ODEPA, 2016.

Regulations to protect the national fauna were put into effect in 2005 and as part of this initiative, the Agricultural Service (Servicio Agrícola y Ganadero, SAG) developed a certification scheme for farms dedicated to animal production (National Livestock Official Certification Scheme (PABCO) [71]), as well as a traceability system (Law No. 20358; [70]) that requires a detailed animal inventory and registration of animal transport. In 2015, a total of 149,367 farms were registered, 75% of which were located in the study region [72]. Voluntary participation in the PABCO scheme varied between groups, ranging between 43% of the farms registered in Group IV, to only 7% in Group II. In turn, this program complements initiatives aimed at the export of quality beef products by facilitating public-private collaboration. Beef exports varied widely between years, and reached a nadir of 2000 ton in 2013 due to weaknesses in the control system (animal identification and control of veterinary inputs) that impeded exports to the EU [73], which requires a detailed registry of animals in real time [74]. More recently, exports rose to 5736 ton in 2015 [14] (Figure A2). Nevertheless, direct sales of producers to exporters occur in only 4% of farms (Table S2). Given the relatively long and cumbersome chains of production, blamed by farmers for low profitability of beef systems (Table 5), more expedient and shorter links between both ends of the production chain seem advisable. Nevertheless, only 5% of family farms had accessed export markets in 2007 [7].

### 4.1. Hypothesized Reason for Change Strategies

Promotion of record keeping and traceability should be complemented with efforts to increase the economic and environmental efficiency of pastoral systems [75]. The adoption of a typification scheme for beef carcasses across the MERCOSUR common market that considers animal breed, age, and farming systems would aid in making available products of known origin and quality for consumers and avoid the competition with beef cuts of unknown traits [76]. Furthermore, a significant increase in the national cattle herd will be required if the aim of the country is to achieve competitive volumes of beef for export. In Group I in particular, there is a need to increase the proportion of beef breeds through genetic improvement, an initiative currently restricted to very small producers [77] where social impact is large but with little impact on overall beef production. Farms in Groups I and II would benefit from focusing on specific market niches that support fair prices, sustainable production and integrated or organic production practices that are compatible with a sustainable intensification. Nevertheless, the small volume of production of these farms, and their focus on cow-calf operations would only make sense if they can enter into strategic alliances with stocker and finishing type of farms that share the same approach to production. Thus, the strengthening of communication networks, via internet, or the more frequent links via regional and local radios, would be essential for this type of approach that could potentially integrate small farmers with slaughter and export plants in the production of quality, value-added beef products. A possible scenario is for Group III farms to finish steers, while Groups I and II provide calves and stockers, an approach that could potentially increase resource use efficiency and a more closely linked, and shorter, chain of production [78]. In view of climate change, constraining beef cattle to native pastures supplemented with crop byproducts and annual forages may be a mitigation strategy and deserves attention in the design of government policies [79,80].

The larger farms in Group IV should emphasize pastoral production prioritizing native pastures with strategic grazing of improved pastures. This approach should be associated with positive externalities, such as the provision of ecosystem services [62] conservation and enhancement of a diverse landscape, and animal wellbeing as reported for the US [81], and for several Latin Americana countries [82]. Furthermore, well managed grasslands may contribute to mitigate the effects of climate change, given their potential to sequester carbon [83]. They should direct their products to markets that value ecosystem and landscape conservation, and that demand natural beef from grazing lands [84]. In this context, the use of supplementary forages should decrease given the high opportunity cost of land [60,79], and they should concentrate on seasonal production of beef, adjusting animal demands to seasonal grassland growth. The wide spread adoption of traceability, and perhaps future payments for ecosystem services, would contribute to the financial performance of these systems as well as to their adaptation to the lower expected availability of rainfall (effect of climate change,) and possible changes in policies, through obtaining higher total revenues that allow the continuity of farms despite obtaining lower productivity per unit area. For example, in these systems there is ample room for the creation of local brands, denomination of origin and equivalent tools for differentiation of quality products [78].

A recent inter-country study that included Chile [85], showed that unless small farmers intensify and increase the efficiency of use of labor and physical resources, and particularly that of grasslands, they may drop out of the beef business.

Resilience and robustness of farms, characteristics that depend on the internal structure and typology of farms, condition adaptation to drivers of change. The number of activities and their mutual match, farm size and availability of resources, and available technologies among others, are associated with resilience and robustness of cattle systems [6,86,87]. The continued existence of beef systems in the region may require the implementation of at least two strategies. First, if a production philosophy based on sustainable intensification is promoted, mixed production systems like those of Groups I and II would benefit from planned crop-pasture rotations, integrated pest management, precision agriculture and enhanced nutrient cycling [88,89,90,91]. Government efforts should address technology transfer, including training on farm management, and incentives to further promote farmers’ associations such as the existing “groups of technology transfer” (GTT and PROFOS) or small farmers enterprises [92,93]. Admittedly, the role of extension services, both public and private, in promoting sustainable intensification of the ruminant livestock sector is unclear, and at least in some countries, there are doubts about their efficiency [94], whereas there is little doubt on the power of demand in shaping farm level production. If this is the case, a much better integration of agroindustry and producers is required, particularly in the case of small cattle producers. The present results show clearly a very limited reach of internet and other information venues that would be essential if the sector is to intensify and raise the quality of its products. The services sector should therefore be encouraged to overcome these limitations [95].

In summary, the changes in input and output prices and the competition with cheaper imported beef cuts appear to have led to a substantial number of changes in the sector, including the increasing sophistication of marketing venues, the improvement of cattle genetics via the introduction of European-type breeds at the expense of the use of dairy calves, the increasing use of certification schemes (traceability and PABCO), and the rise in use of government credits for pasture reclamation. Nevertheless, larger farms and more intensively managed medium properties may have been favored by these changes.

### 4.2. Future Challenges for the Beef Sector

Beef plays an important role in the diet of Chile’s population (24 kg per capita in 2015). The country has a very high incidence of infant and adult obesity [96], and this is particularly noticeable in urban environments, where unhealthy, convenience, low-priced foods are widely consumed. The current labeling regulations in Chile warn against unhealthy foods but do little to educate people in positive terms. The role of meats and beef, in particular, in this setting is uncertain as the population has been exposed to contradictory information in the media. Therefore, a strengthening of the rural-urban beef chain would require policies aimed at clarifying the roles of beef in healthy diets since urban consumption patterns are major drivers of changes in the rural food sector [94,97], actions that should involve retailers and the food services sector as well as the public sector [98].

In summary, the long-term existence and persistence of grassland-based, sustainable beef production in southern Chile relies on a combination of technologies and knowledge to adapt production to local conditions, and to a larger extent, on the development and implementation of a long-term plan that integrates enabling policies into a coherent whole.

## 5. Conclusions

Cattle producing farms were shown to be dynamic, evolving in response to market forces and government policies and support. Their structure and size influenced their resilience and robustness. Larger farms with access to technical advice, using a variety of management practices, and involved in short chains of production, showed less variability than the smaller properties. Cropping activities and various crop byproducts provided flexibility in land use in all farms.

Due to the small number of farmers that export their production, the incentives of the government Operational Plan for Beef Exports influenced a small number of producers. To achieve its stated objectives, a more holistic approach is required, and in particular, much closer interactions within-and between producers and retailers, and shorter and more effective links along the production chain are required to align production strategies with government policies and objectives. Initiatives such as wider implementation of traceability and participation in the PABCO initiatives should be stimulated, but this will require an important effort for online, real time, record keeping. Formal association and cooperation between producers and industry should permit a fluid, and more profitable, movement of calves and stockers to farms specializing in fattening of steers.

Small and medium farms in the study region face many of the same challenges reported for other Mediterranean countries in Europe, and there is therefore an opportunity to exchange information regarding development strategies, market intelligence and technologies.

The farm groups identified in the present study will need to follow different development paths for their survival. Extensive systems may have a competitive advantage in market niches that demand quality and “natural animal products”, associated with perceived positive ecosystem services and landscape conservation. These characteristics fit well with Group IV farms. The smaller farms in Groups I and II need to pursue a strategy of sustainable intensification via improved management of animal and natural resources. It is suggested that smaller farms should build commercial bridges with farms in Group III to complete the production cycle, assuming that they share the same production philosophy.

A concerted effort between farmers and government is required with regard to the role of ruminant products in healthy human diets, an issue that is presently confounded by misinformation, conflicting commercial interests and frequent ignorance in the media. The sector should therefore promote its natural, grass-fed, ruminant products coming largely from labor intensive and diverse family farms.

It is anticipated that the effect of climate change in the region that is associated with significantly reduced rainfall, will lead to lower grassland and crop production. Farmers will therefore face increased challenges in managing their resources and adopting new technologies, while government policies will have to evolve if small and medium family farms are to subsist. These changes will likely be the main drivers of cattle production systems and will demand continued adaptation strategies by both the public and private sectors.

## Figures and Tables

**Table 1:** Quantitative (mean ± standard deviation) and qualitative variables (%) of beef production systems in the Southern Zone of Chile (2009–2015).

Variables Characterization		2009	2011	2013	2015	Total
Number of farms		934	998	1111	1120	4163
Total cattle, head of cattle		367.6 ± 505.5 ^d^	342.5 ± 549.3 ^c^	263.2 ± 522.1 ^b^	214.1 ± 440.4 ^a^	292.4 ± 508.1
Stocking rate, LU·ha^−1,†^		0.83 ± 0.68 ^b^	0.72 ± 0.65 ^a^	0.7 ± 0.65 ^a^	0.72 ± 0.61 ^a^	0.74 ± 0.65
Surface area, ha		540 ± 1100 ^c^	611 ± 1177 ^c^	492 ± 1159 ^b^	409 ± 913 ^a^	509 ± 1091
Sown forages, % total surface área ^#^		14.9 ± 20.3 ^b^	8.9 ± 17.9 ^a^	7.0 ± 15.0 ^a^	6.4 ± 15.3 ^a^	9.1 ± 17.4
Improved pastures, % total surface área ^#^		21.8 ± 26.4 ^b^	22.5 ± 29.8 ^b^	14.7 ± 25.6 ^a^	14.7 ± 25.9 ^a^	18.2 ± 27.2
Native pastures, % total surface área ^#^		19.5 ± 26.6 ^a^	23.6 ± 27.8 ^b^	35.4 ± 30.3 ^c^	41.4 ± 34.1 ^d^	30.6 ± 31.2
Supplementary crops, %total surface área ^#^		5.0 ± 10.6 ^b^	1.5 ± 5.3 ^a^	1.7 ± 5.9 ^a^	1.3 ± 5.3 ^a^	2.3 ± 7.1
Other agricultural uses, %total surface área ^#^		38.8 ± 29.0 ^b^	43.4 ± 28.8 ^c^	41.3 ± 29.5 ^b,c^	36.2 ± 29.7 ^a^	39.9 ± 29.4
Beef breeds, % total Cattle		62.3 ± 43.0 ^b,c^	63.3 ± 43.0 ^c^	47.0 ± 44.1 ^a^	58.6 ± 44.0 ^b^	57.5 ± 44.3
Other crosses or beef breeds, % total cattle		53.6 ± 47.2 ^b^	53.9 ± 47.0 ^b^	26.9 ± 43.1 ^a^	38.9 ± 47.0 ^a^	42.6 ± 47.4
Measurement of forage availability, % total farms		12.0	7.2	6.9	11.6	9.4
Soil fertility analysis, % total farms		65.0 *	40.7 *	31.9 *	64.0 *	50.1
Electric fence, % total farms		66.7 *	44.6 *	42.0 *	62.3	53.6
% cattle sold per sales channel,	Auction	72.0 *	69.8 *	53.8	42.3 *	58.2
	Private Fair	5.8	6.7	0.0 *	3.3	3.7
	Livestock dealer	9.2	11.2	13.8	6.4	10.0
	Dealers	9.6 *	4.4 *	25.7	37.6 *	20.9
	Slaughterhouse	3.4	3.6	6.3	10.4 *	6.2
	Other (Direct export)	0.0	4.3 *	0.3	0.0 (0.62)	1.0
Crops as the main or only source of income, % total farms		47.3	53.8	68.4 *	59.9	40.7
Investments, % total farms	Purchase first-calf heifers	0.1 *	5.0	4.8	2.9	3.2
	Pastures	0.3 *	52.0	32.8	47.8	33.6
	Infrastructure and machinery	12.4	13.9	0.0	8.1	8.3
	Others	26.3 *	8.2	0.0 *	2.1 *	8.6
	No investment	60.8 *	20.9 *	62.4 *	39.2	46.3
Agronomic advice, % total farms		…	25.4	36.6	33.6	32.1
Veterinary advice, % total farms		…	51.1	46.1	51.0	49.3
Management advice, % total farms		…	6.3	9.9	11.4	9.3
PABCO subscribers, % total farms **		…	…	16.5	15.5	16

* Years with averages that differ significantly from the expected value (*p* < 0.05); ^a–d^ Within row, averages with different superscript differ significantly (*p* < 0.05); ^†^ Livestock Unit equivalent to 500 kg of live weight; ^#^ Corresponds to the percentage of the total area or total of animals that fall within the category described; ** National Livestock Official Certification Scheme.

**Table 2:** Comparison of characterization variables between groups (mean ± standard deviation).

Variables	Group I	Group II	Group III	Group IV
Farms, % total farms (Number)	59 (2453)	14 (580)	17 (698)	10 (432)
Dimension				
Total cattle, nº	117.4 ± 146.9 ^a^	112.9 ± 127.6 ^a^	465.7 ± 511.48 ^b^	1247.4 ± 863.8 ^c^
Beef steers, nº	12.1 ± 38.4 ^a^	10.0 ± 28.3 ^a^	330.0 ± 424.4 ^c^	228.2 ± 389.9 ^b^
Cows, nº	45.4 ± 67.3 ^b^	47.6 ± 58.7 ^c^	12.2 ± 35.6 ^a^	409 ± 362.7 ^d^
Breeding cows, nº	43.1 ± 66.2 ^b^	45.5 ± 57.6 ^b^	8.9 ± 30.0 ^a^	380.7 ± 358.9 ^c^
Heifers, nº	19.8 ± 37.4 ^a^	16.4 ± 23.7 ^a^	36.4 ± 87.7 ^b^	234.8 ± 250.1 ^c^
Breeding heifers, nº	13.7 ± 27.4 ^a^	13.6 ± 20.8 ^a^	9.7 ± 43.4 ^a^	154.0 ± 193.6 ^b^
Surface area, ha	301.0 ± 462.5 ^a^	329.0 ± 397.1 ^a^	372.7 ± 384.0 ^b^	2153.8 ± 2607.2 ^c^
Native pastures, ha	81.9 ± 226.2 ^a^	101.7 ± 168.8 ^a^	61.1 ± 156.6 a	439.5 ± 12205 ^b^
Improved pastures, ha	26.8 ± 62.7 ^a^	16.5 ± 41.0 ^a^	128.5 ± 178.3 ^b^	274.3 ± 350.1 ^c^
Sown pasture, ha	15.8 ± 45.6 ^a^	13.1 ± 46.7 ^a^	58.7 ± 113.8 ^b^	161.3 ± 267.6 ^c^
Supplementary crops, ha	4.3 ± 15.8 ^a^	3.8 ± 12.8 ^a^	11.1 ± 27 ^b^	48.8 ± 88.0 ^c^
Corn for silage, ha	0.5 ± 6.2 ^a^	0.3 ± 2.9 ^a^	3.4 ± 15.5 ^b^	9.3 ± 32.1 ^c^
Other agricultural uses, ha	172.8 ± 334.6 ^a^	195.3 ± 297.2 ^a^	116.3 ± 216.9 ^a^	1236.5 ± 2265.0 ^b^
Labor, Nº permanent workers	1.0 ± 3.1 ^a^	1.1 ± 2.0 ^a^	2.5 ± 4.1 ^b^	6.1 ± 11.1 ^c^
Use of resources				
Native pastures, % total surface area	33.5 ± 30.7 ^c^	42.4 ± 32.7 ^d^	18.8 ± 29.5 ^a^	17.4 ± 23.9 ^b^
Improved pastures, % total surface area	14.4 ± 24.7 ^a^	7.7 ± 16.5 ^a^	37.1 ± 33.1 ^c^	23.5 ± 26.0 ^b^
Sown pastures, % total surface area	7.3 ± 15.6 ^a^	4.8 ± 11.8 ^a^	15.9 ± 22.4 ^b^	13.6 ± 20.0 ^b^
Total pastures, % total surface area	55.2 ± 29.1 ^b^	54.6 ± 30.5 ^a^	71.8 ± 26.0 ^d^	54.6 ± 28.5 ^b^
Supplementary crops, % total surface area	1.8 ± 5.6 ^a^	1.1 ± 3.1 ^a^	2.7 ± 6.5 ^b^	5.9 ± 14.7 ^c^
Corn for silage % total surface area	0.3 ± 2.2 ^a^	0.2 ± 1.4 ^a^	1.1 ± 4.6 ^b^	1.5 ± 6.8 ^b^
Other agricultural uses, %total surface area	43.0 ± 29.2 ^c^	44.3 ± 30.6 ^d^	25.5 ± 25.0 ^a^	39.5 ± 28.3 ^b^
Cows, % total Cattle	42.2 ± 21.8 ^c^	45.0 ± 17.0 ^c^	3.8 ± 8.7 ^a^	35.6 ± 20.4 ^b^
Breeding cows, % total cattle	40.2 ± 22.3 ^c^	43.6 ± 18.0 ^d^	2.9 ± 7.7 ^a^	33.1 ± 20.9 ^b^
Heifers, nº, % total Cattle	15.9 ± 18.1	13.9 ± 11.9	9.3 ± 18.5	19.5 ± 17.6
Breeding heifers, nº, % total Cattle	12.0 ± 13.9 ^b^	12.2 ± 10.5 ^b^	3.0 ± 10.4 ^a^	13.3 ± 14.5 ^b^
Males, % total cattle	9.7 ± 13.8 ^a^	8.4 ± 10.9 a	70.8 ± 31.4 ^b^	18.3 ± 22.4 ^c^
Beef steers, % total cattle	7.6 ± 14.1 ^a^	6.1 ± 11.0 a	70.5 ± 31.6 ^c^	16.7 ± 22.9 ^b^
Beef breeds, % total cattle	40.0 ± 43.2	94.4 ± 13.3	72.7 ± 38.1	82.5 ± 30.8
Overo Colorado, % total cattle	57.3 ± 45.6 ^b^	10.8 ± 30.5 ^a^	26.1 ± 40.3 ^a^	28.3 ± 43.6 ^a^
Angus, % total cattle	23.4 ± 39.6 ^b^	3.6 ± 12.9 ^a^	16.3 ± 33.1 ^b^	42.1 ± 43.0 ^c^
Hereford % total cattle	11.6 ± 29.3 ^c^	1.1 ± 7.2 ^a^	5.4 ± 17.6 ^b^	7.5 ± 18.6 ^b^
Other crosses or beef breeds, % total cattle	23.1 ± 40.4	95.3 ± 15.0	65.7 ± 45.4	45.7 ± 44.8
Labor, Permanent workers·100 bovines^−1^	2.9 ± 5.0 ^c^	0.7 ± 1.2 ^a^	1.1 ± 2.0 ^b^	0.9 ± 1.3 ^b^
Stocking rate, LU·ha^−1,^^†^	0.65 ± 0.59 ^a^	0.59 ± 0.53 ^a^	1.02 ± 0.73 ^b^	1.02 ± 0.75 ^b^

^a–d^ Within-row averages with different superscripts differ significantly (*p* < 0.05); ^†^ Livestock Unit equivalent to 500 kg of live weight.

**Table 3:** Principal components, eigenvalue, explained and accumulated variance and correlation coefficients of the variables with principal components.

Principal Component	Eigenvalue	% Explained Variance (% Cumulative Variance)	Variables	Correlation
1	4.1	20.6 (20.6)	Cows, % total cattle	−0.89
			Beef steers, % total cattle	0.90
			Males, % total cattle	0.91
			Breeding cows, % total cattle	−0.87
2	3.4	17.2 (37.8)	Improved pastures, ha	0.71
			Cows, nº	0.80
			Heifers, nº	0.76
			Total cattle, nº	0.87
			Surface area, ha	0.55
3	2.5	12.5 (50.3)	Supplementary crops, ha	0.85
			Supplementary crops, % Total surface area	0.85
			Fodder oats, ha	0.63
			Annual ryegrass, ha	0.57
			Other supplementary crops, ha	0.76
4	1.8	8.9 (59.2)	Total pastures, % Total surface area	−0.67
			Stocking rate, LU·ha^−1,†^	−0.68
			Other agricultural uses, ha	0.79
			Surface area, ha	0.72
5	1.4	7.6 (66.3)	Other crosses or beef breeds, % Total cattle	0.83
			Labor, Permanent workers·100 bovines^−1^	−0.41
			Beef breeds, %	0.79

^†^ Livestock Unit equivalent to 500 kg of live weight.

**Table 4:** Comparison of characterization variables between groups (%).

Variables		Group I	Group II	Group III	Group IV
Measurement of forage availability, % total farms		5.3 *	5.3 *	14.9	28.9 *
Soil fertility analysis, % total farms		42.3 *	61.2	59.2	64.6
Electric fence, % total farms		46.2 *	57.6	68.5 *	66.4
Sales Channel, % cattle sold	Auction	58.4	49.8	66.4 *	54.4
	Private Fair	2.8	1.9	7.2	5.7
	Livestock tradesman	8.4	11.0	8.4	20 *
	Dealers	26.5 *	32.6 *	3.6 *	3.6 *
	Slaughterhouse	3.3 *	3.7	13.0	14 *
	Other (Export direct)	0.6	0.9	1.5	2.4
Agricultural as the main or only source of income, % total farms		59.7	54.3	56.5	56.5
Investments, % total farms	Purchase first-calf heifers	3.6	1.7	1.4	5.5
	Pastures	31.3	37.3	32.6	42.8
	Infrastructure and machinery	6.5	8.7	11.5	12.8
	Others	8.1	9.0	10.0	8.6
	Without investment	50.5 *	43.3	44.6	30.2 *
Agronomic advice, % total farms		24.3	18.6	22.8	40 *
Veterinary advice, % total farms		34.7	33.6	40.5	61.1 *
Management advice, % total farms		5.5	9.8	6.3	15 *
PABCO subscribers, % total farms ^†^		12.2 *	6.7 *	29.2	42.8 *
Internet access in farms (data only 2015)		13.9 *	7.5 *	38	61 *
Use internet in farms activities (data only 2015)		17.7 *	11.3 *	51.0 *	80.7 *

^†^ National Livestock Official Certification Scheme.; * Years with averages that differ significantly from the expected value (*p* < 0.05).

**Table 5:** Causes associated with the decrease in the number of animals per group.

Variables	Groups				Total
	I	II	III	IV	
Farms with decreasing herd size, % total farms	40.4	39.3	29.1	21.6 ^*^	37.1
Causes of decreased herd size					
Change of land use	4.2	9.6 *	0.0 *	5.3	5.3
Low profitability	19.7	32.2	22.7	26.3	23.8
Lack of marketing channels	0.4	1.7	4.5 *	0.0	1.2
Animal theft	2.1	9.6 *	0.0 *	0.0 *	3.8
Lack of capital	10.1	1.7 *	9.1	5.3	7.5
Drought or lack of water	29.8	18.3 *	31.8	36.8 *	27.2
Producer’s disease	11.3	7.8	4.5	5.3	9.4
Other	22.3	19.1	27.3	21.1	21.9

* Years with averages that differ significantly from the expected value (*p* < 0.05).

## Supplementary Materials

The following are available online at https://www.mdpi.com/2076-2615/9/12/1135/s1. Table S1: Comparison of quantitative variables by survey year within group (mean ± standard deviation), Table S2: Comparison of qualitative variables by survey year within group.

## Appendix A

**Table A1:** Selected questions of bovine livestock survey.

**SECTION IV. SOIL USE AND PASTURES MANAGEMENT**					
Soil use (to the day of the survey)			Supplementary crops (last 12 months)		
	Area				Area
Sown pasture			Forage oats		
Improved pastures			Ryegrass annuals, biennials and short rotation		
Native pastures			Corn for silage		
Supplementary crops			Forage Raps		
Other uses			Forage barley		
Total surface area			Forage turnip	Winter	
				Summer	
			Other	Indicate which: _________	
			Total surface area		
Pastures management (last 12 months)					
Use electric fence		1. Yes	2. No		
Performs soil fertility analysis		1. Yes	2. No		
Performs weed control		1. Yes	2. No		
Use slurry as prairie fertilizer		1. Yes	2. No		
Measure forage availability		1. Yes	2. No		
					
Type of grazing		1. Rotative	2. Continuous	3. Estabulated or feedlot (does not graze)	
**SECTION V. EXISTENCES**					
TOTAL EXISTENCE OF BOVINE TO THE DAY OF THE SURVEY			EXISTENCE OF BOVINE BY BREED TO THE DAY OF THE SURVEY		
Record own and foreign animals in the farm		Number			Number
Cows			Overo negro	Predominantly european	
Heifers (females older than 1 year old)				Predominantly american	
Calves (females under 1 year old)			Overo Colorado		
Calves (males under 1 year old)			Jersey		
Steers (castrated males 1 year and older)			Crosses for milk		
Little bulls (whole males 1 year and older)			Other breeds for milk ____________		
Bulls			Angus		
Oxen			Hereford		
Total			Crosses for meat _________________		
			Other breeds for meat ___________		
EXISTENCE OF BEEF CATTLE TO THE DAY OF THE SURVEY		Number	Total		
Cows	Breeding				
	Fattening				
	Cull cow				
Heifers	Breeding				
	Fattening				
Calves (females under 1 year old)					
Calves (males under 1 year old)					
Steers (castrated males 1 year and older)					
Bulls (in service and breeding)					
Oxen					
**Total**					
